# 

**Published:** 1903-05-02

**Authors:** 


**Notices of Births, Deaths, and Marriages** are inserted in this column at a charge of 2s. 6d. for an announcement not exceeding four lines.

**MARRIAGE.**

Pallot—Reynolds.—April 22nd (by special license), at St. Saviour's Church, St. Heliers, Jersey, by the Rev. Luce, Charles Pallot, of the Maison, Mathias St. Servan, Brittany, to Marian Emily, fourth daughter of John and Harriett Reynolds, Falfield, Gloucestershire. (1143)

### NOTICE TO CORRESPONDENTS.

All MSS., Letters, Books for Review, and other matters intended for the Editor should be addressed The Editor, "The Hospital," The Hospital Buildings, 28 & 29 Southampton Street, Strand, W.C.

The Editor cannot undertake to return rejected MS., even when accompanied by stamped directed envelope.

**Notice to Advertisers and Subscribers.**

All Advertisements, Orders for Copies of The Hospital, and Business Communications should be addressed to **THE MANAGER, (Not to the Editor)**, The Hospital, 28 & 29 Southampton Street, Strand, London, W.C.

The Cost of Subscription, post free, is as follows.—Per week, 3½d.; per quarter, 3s. 9d.; per half-year, 7s. 6d.; per annum, 15s. Foreig and Colonial:—Per annum, 19s. 6d., payable, in all cases, in advance.

**NOTICE.—Vol. XXXIII. of "The Hospital" (October 1902 to March 1903) in handsome cloth binding, will shortly be on sale at the office of this Journal, price 7s. 6d. Cases for binding the Numbers contained in this Volume 1s. 6d. Index to Vol. XXXIII., 2½d., post free.**

**The monthly part for May is now ready. Price 1s., by post 1s. 3d.**

### BOOKS RECEIVED.

Hodder and Stoughton.

"Nerves in Disorder." By A. T. Schofield, M.D.

J. and A. Churchill.

"Ocular Therapeutics." By Davies and Stephenson.

G. E. Stechert.

"The Manual Treatment of Diseases of Women." By Gustaf Norström, M.D.

"Chronic Headache and its Treatment by Massage." By Gustaf Norström, M.D.

### The Student of Medicine.

### APPOINTMENTS.

**Donald John Armour**, B.A., M.B.Tor., M.R.C.P.Lond., F.R.C.S.Eng., appointed Assistant Surgeon to the West London Hospital. **James Bannerman**, M.B., C.M.Edin., appointed Medical Officer of Health for Stanhope Rural District, and Medical Officer and Public Vaccinator for Stanhope District of Weardale Union. **Wm. Brander**, M.B., Ch.B.Aberd., appointed Resident Medical Officer to the Ecclesall Bierlow Union Infirmary, Sheffield. **H. S. Brightmore**, M.B., Ch.B.Vict., appointed District Medical Officer of the Salford Union. **Josephine Brown** M.B.Lond., appointed Assistant Medical Officer to the Lincoln County Asylum, Bracebridge. **W. Brodie Brown**, M.B., C.M. Aberd., appointed Medical Officer and Public Vaccinator for Aboyne and Glentanner. **D. R. Powell Evans**, L.R.C.P.Lond., M.R.C.S.Eng., L.S.A.Lond., appointed Clinical Assistant to the Samaritan Hospital for Women. **G. B. Forge**, M.R.C.S.Eng., L.R.C.P.Lond., appointed District and Workhouse Medical Officer of the Rochford Union. **James Harrison**, M.R.C.S.Eng., L.R.C.P.Edin., appointed Medical Officer for the Workhouse and First District of the East Grinstead Union. **W. F. Haynes**, L.R.C.P.Lond., appointed District Medical Officer of the Bishop Stortford Union. **T. Barrett Heggs**, M.B., Ch.B., appointed House Physician to the Sussex County Hospital. **H. W. Hodgson**, M.R.C.S.Eng., L.R.C.P.Lond., appointed Second Assistant Medical Officer of Whitechapel Union. **Robert Jardine**, M.D.Edin., M.R.C.S.Eng., L.F.P. and S.Glasg., F.R.S.Edin., appointed Professor of Midwifery in St. Mungo's College, Glasgow. **G. A. Johnston**, M.D.Brux., F.R.C.S., L.R.C.P.I., appointed District Medical Officer of the Kendal Union. **Harold Kerr**, M.B., Ch.B.Edin., appointed Assistant House Surgeon to Rotherham Hospital and Dispensary. **E. H. Kitchin**, M.B., B.C.Cantab., appointed Medical Officer of the Skipton Union Workhouse. **George Knapton**, L.R.C.P.Edin., etc., appointed Medical Examiner for the Scottish Imperial Insurance Company. **James Mackay**, M.B.Aberd., etc., appointed Medical Examiner for Hearts of Oak Benefit Society for Manchester, Moss Side, and Old Trafford. **Alex. MacLennan**, M.B., C.M., L.M., appointed Visiting Surgeon to the Glasgow Training Home for Nurses. **Denis Nyhan**, L.R.C.P., L.R.C.S.Edin., L F.P.S.Glasg., appointed Medical Officer of Health to the Brynmawr District Council. **F. W. Perry**, L.R.C.P., L.R.C.S.I., appointed District Medical Officer to the Malling Union. **A. M. Toms**, M.R.C.S.Eng., L.S.A., appointed District Medical Officer of the Tenterden Union. **J. D. Wardale**, M.B., appointed Lecturer on Ophthalmology in the University of Durham College of Medicine, Newcastle-upon-Tyne. **T. R. C. Whipham**, M.B., B.Ch.Oxon., M.R.C.P.Lond., appointed Physician to Outpatients to the Evelina Hospital for Sick Children. **J. B. White**, M.D., M.Ch., R.U.I., appointed Medical Officer to the Homerton Workhouse of the City of London Union.

### BOOKS THAT NURSES NEED.

**Do you want to be a Nurse?**

### THE NURSING PROFESSION: HOW AND WHERE TO TRAIN

Will give you all the information you require.

Price 2/4 post free.

**When you are a Probationer you will need—**

### THE NURSE'S DICTIONARY of MEDICAL TERMS.

By Honnor Morten. Price 2/- post free.

### NURSING: ITS THEORY and PRACTICE.

By Percy Lewis, M.D. Price 3/6 post free.

**When you are a Staff Nurse you will need—**

### A HANDBOOK FOR NURSES.

This is the most advanced text-book published for Nurses.

By J. K. Watson, M.D. Price 5/4 post free.

### SURGICAL WARD-WORK and NURSING.

By Alexander Miles, M.D. 3/10 post free.

**If you desire to be proficient in all branches of Nursing you will need—**

### FEVERS AND INFECTIOUS DISEASES.

By William Harding, M.D. Price 1/- post free.

### GYNÆCOLOGICAL NURSING.

By G. Hawkins-Ambler, F.R.C.S. Price 1/- post free.

### NURSING in DISEASES of the THROAT, NOSE, and EAR.

By P. M. Yearsley, F.R.C.S. Price 2/6 post free.

### OPHTHALMIC NURSING.

By Sydney Stephenson, F.R.C.S. Price 3/10 post free.

### ART OF MASSAGE.

By A. Creighton Hale. Price 6/- post free.

All these books can be obtained at all Booksellers', and at THE SCIENTIFIC PRESS, 28 Southampton Street, Strand, W.C., who publish or sell every kind of Text-book for Nurses.

